# Gene set analysis exploiting the topology of a pathway

**DOI:** 10.1186/1752-0509-4-121

**Published:** 2010-09-01

**Authors:** Maria Sofia Massa, Monica Chiogna, Chiara Romualdi

**Affiliations:** 1 Department of Statistical Sciences, University of Padova, via C. Battisti 241, Padova, Italy; 2 Department of Biology, University of Padova, via U. Bassi 58/B, Padova, Italy

## Abstract

**Background:**

Recently, a great effort in microarray data analysis is directed towards the study of the so-called gene sets. A gene set is defined by genes that are, somehow, functionally related. For example, genes appearing in a known biological pathway naturally define a gene set. The gene sets are usually identified from a priori biological knowledge. Nowadays, many bioinformatics resources store such kind of knowledge (see, for example, the Kyoto Encyclopedia of Genes and Genomes, among others). Although pathways maps carry important information about the structure of correlation among genes that should not be neglected, the currently available multivariate methods for gene set analysis do not fully exploit it.

**Results:**

We propose a novel gene set analysis specifically designed for gene sets defined by pathways. Such analysis, based on graphical models, explicitly incorporates the dependence structure among genes highlighted by the topology of pathways. The analysis is designed to be used for overall surveillance of changes in a pathway in different experimental conditions. In fact, under different circumstances, not only the expression of the genes in a pathway, but also the strength of their relations may change. The methods resulting from the proposal allow both to test for variations in the strength of the links, and to properly account for heteroschedasticity in the usual tests for differential expression.

**Conclusions:**

The use of graphical models allows a deeper look at the components of the pathway that can be tested separately and compared marginally. In this way it is possible to test single components of the pathway and highlight only those involved in its deregulation.

## Background

A microarray experiment typically provides a list of differentially expressed genes that represents the starting point of a highly difficult process of results interpretation. Biological interpretation becomes easier if differentially expressed genes show some similarity according to their functional annotation. Thus, in recent years, the interest has moved from the study of individual genes to that of groups of genes (defined by functional categories or metabolic pathways) and methods for gene set analysis have received a great attention. The aim is to identify groups of genes with moderate, but coordinated, expression changes, which should enable the understanding of cellular processes involved in the biological problem at hand. Such approaches directly score pre-defined gene sets for differential expression.

Several gene set analysis methods have been recently developed, both in the univariate and multivariate context. [1] divide gene sets methods into two broad categories: (i) methods based on enrichment analysis performed on a list of genes selected through a gene-level test; (ii) methods based on global and multivariate approaches that define a model on the whole gene set. For a comprehensive review on existing methods see [1] and references therein. For a detailed description and for a critical investigation of the hypothesis tested in both approaches see [2-5].

The main concerns with the first class of methods are the assumption that genes are independent, and the use of a cut-off threshold value for the selection of differentially expressed genes. Indeed, [6] show that the final result of these approaches is significantly affected by the selected threshold, which is normally chosen arbitrarily. In this way, many genes with moderate but meaningful expression changes are discarded by the strict cut-off value, which leads to a reduction in statistical power. On the other hand, global and multivariate approaches relax the assumption of independence among genes belonging to the same gene sets and identify moderate, but coordinated, expression changes that cannot be detected by the previous approach [3].

In the multivariate perspective, [2] propose Global Test, modelling differential gene expression by means of random-effects logistic regression models, while [7] propose ANCOVA Global Test, which is similar to Global Test but with phenotype and genes exchanged in regression models. ANCOVA Global Test seems to outperform Global Test, especially in cases where the asymptotic distribution of Global Test cannot be used [7]. More recently, [8] propose a MANOVA test using a shrinkage covariance matrix estimator for the sample covariance matrix.

One of the databases widely in use for the a priori definition of gene sets is the Kyoto Encyclopedia of Genes and Genomes (KEGG in the following) [9], where gene products are structured into several known metabolic and regulatory pathways. A pathway is a graphical diagram of biochemical reactions involving different enzymes, where directed and undirected edges connect few different gene products at time, according to their chemical interactions. Although KEGG pathways are usually applied to define gene sets, the approaches so far proposed do not explicitly take into consideration the dependence structure among genes implied by the topology of the pathway.

We propose to pursue the study of the behaviour of pathways in different experimental conditions within a graphical models context. This approach, whose application in the context of pathways analysis is still largely unexplored, goes in a direction which can valuably complement approaches more extensively offered by the current literature. In fact, by recording the structure of the pathway in an appropriately defined graph, we are able to keep track of the biochemical structure and reactions of the enzymes. In taking this route, the main interest is not on the detection of the structure of the pathway, because we consider it as fixed from the very beginning. In this sense, our approach differs from approaches for the analysis of differential coexpression [10]. In other words, we are not interested in learning the structure of the pathways from the data (see [11]); instead, we exploit the available biological knowledge to define appropriate statistical analyses.

Within the graphical models context, data are considered as coming from Gaussian multivariate distributions with a structured concentration matrix (inverse of the covariance matrix), which reflects dependencies among variables. We present in detail two statistical tests for comparing gene sets under different experimental conditions, which naturally stem from the adopted theoretical framework. The first one addresses the question of testing whether the strength of the connections among genes is altered in different experimental condition. It is likely to figure that a pathological condition does not change the structure of a pathway, but, rather, can influence the strength of the biochemical reactions. For example, a strong partial correlation among two genes in the healthy state could diminish in the disease state, or vice versa. Therefore, discovering any statistically significant difference among conditions that share the same underlying chemical structure is a crucial information. Our first test focusses, in particular, on the strength of the links among gene products and on their possible changes when considering two (or more) experimental conditions of interest. The second test is more traditionally designed for testing for differential expression. In doing the test, we specifically employ the information about the behaviour of the partial correlations among genes and about their possible heteroschedasticity in different experimental conditions. We stress that the two tests can be performed independently one from each other. Of course, if one performs both of them, there is a suggested natural order, but the particular research question will define if they are both necessary or if, in a preliminary phase, only one of them is requested.

The adoption of graphical methods makes it possible to decompose the overall statistical model into smaller models, with the aim of exploring in more detail small portions of the entire model. This ability naturally leads us to wish to compare portions of the pathways, with the aim of identifying subgroups of genes which appear to drive differences (deregulations) of the entire structure. The application of this idea to biological pathways is highly innovative, as it allows to look in detail to components of the pathway, opportunely defined, that can be studied separately. In fact, the expression/correlation behaviour of a large pathway could be misleading, hiding significant parts of the pathway mostly involved in the biological process under exam. With the help of the graphical models arguments, we attempt to uncover such parts.

## Results and Discussion

Two separate datasets have been used to test our model-based approach, both pertaining to gene expression changes derived by specific genetic alterations in cancer.

### Data: BCR signaling pathway

**Figure 1:**
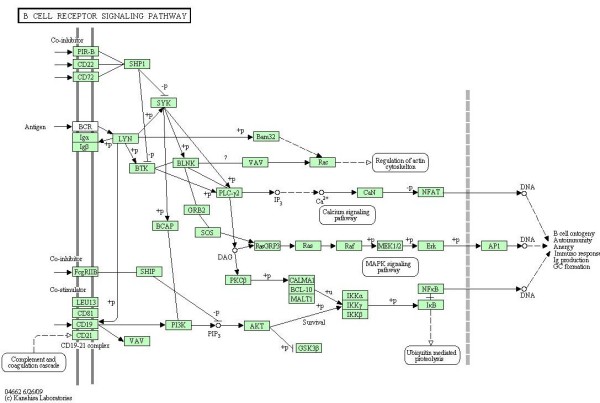
**BCR signaling pathway**. B cell receptor signaling pathway in human taken from KEGG [9].

The first dataset that we use has been recently published by [12], and characterizes gene expression signatures in acute lymphocytic leukemia (ALL) cells associated with known genotypic abnormalities in adult patients. Several distinct genetic mechanisms lead to acute lymphocytic leukemia (ALL) malignant transformations deriving from distinct lymphoid precursor cells that have been committed to either T-lineage or B-lineage differentiation. Chromosome translocations and molecular rearrangements are common events in B-lineage ALL and reflect distinct mechanisms of transformation. The relative frequencies of specific molecular rearrangements differ in children and adults with B-lineage ALL. The B cell Receptor (BCR/ABL) gene rearrangement occurs in about 25% of cases in adult ALL, and much less frequently in pediatric ALL. Because these cytogenetic abnormalities reflect distinct mechanisms of transformation, molecular differences between these two types of rearrangements could help to explain why children and adults with ALL have such different outcomes following conventional therapy. Data are freely available at the Bioconductor web site. Expression values, appropriately normalized according to *rma *and quantile normalization, derived from Affymetrix single channel technology, consist of 37 observations from one experimental condition (*n*_1 _= 37, BCR; presence of BCR/ABL gene rearrangement) and 41 observations from another experimental condition (*n*_2 _= 41, NEG; absence of rearrangement). Gene BCR is central in this study, because it is involved in the process of rearrangement. For this reason, we decide to focus our approach on the B cell receptor signaling pathway (represented in Figure 1), which has the gene BCR as input. If there is a rearrangement of this gene, we expect that also the genes belonging to the connected pathway will be highly influenced from the BCR/ABL gene rearrangement. We consider the whole pathway, that has 35 gene products (*p *= 35). If one enzyme includes multiple gene products, we consider as representative of the enzyme the gene most differentially expressed between the two conditions, according to SAM test [13].

### Data: ERBB signaling pathway

**Figure 2:**
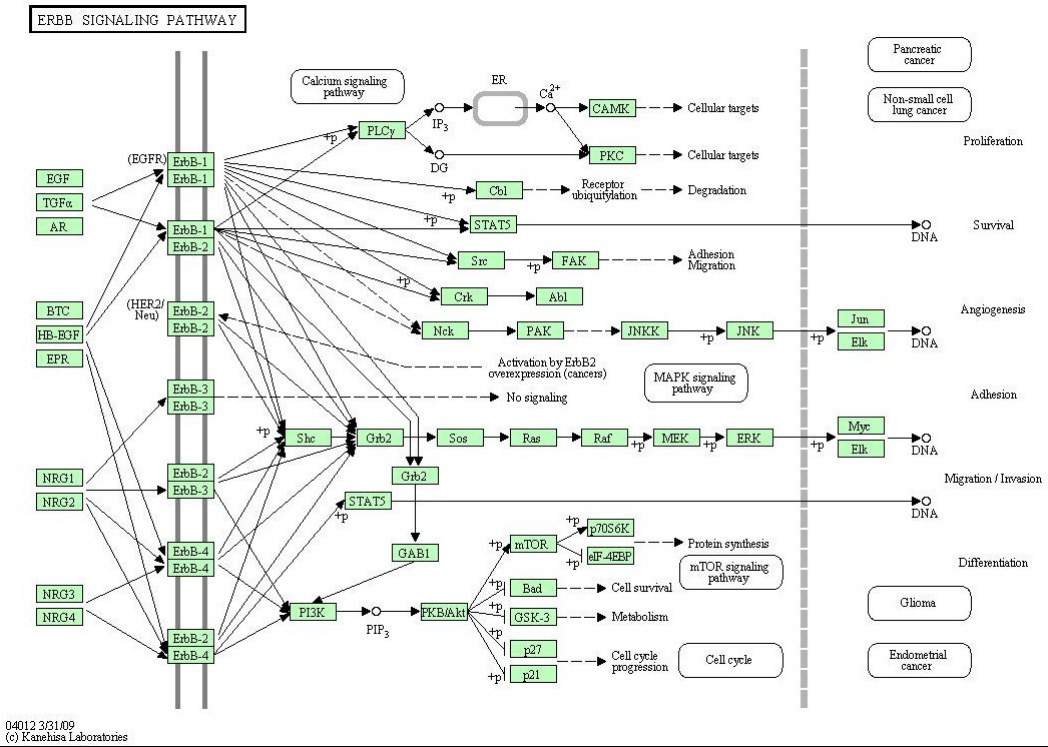
**ERBB signaling pathway**. ERBB signaling pathway in human taken from KEGG [9].

The second dataset published by [14] uses gene expression profiles of 90 patients affected by lung adenocarcinoma to determine the relationship between expression and underlying genetic changes, which are known to be important for the pathogenesis of lung cancers. In particular, the Authors investigate three major targets for genetic changes, p53, epidermal growth factor receptor (EGFR, alias ERBB-1), and K-ras. In particular, EGFR mutations are present in a subset of pulmonary adenocarcinomas, and tumors with this mutation have been shown to be highly sensitive to gefitinib, a drug that inhibits EGFR, selectively targeting proteins in malignant cells. Thus, here we evaluate the difference between patients with (*n*_1 _= 32) and without (*n*_2 _= 58) EGFR mutation focusing on ERBB signaling pathway (reported in Figure 2).

The Authors used a two-channel technology (patients vs. common reference) with a custom Agilent oligonucleotide microarray, containing a total of 21,619 spots corresponding to 18,175 unique genes. Data are freely available at GEO database with GSE11969 ID. For our analyses we use normalized expression values submitted in the database. ERBB signaling pathway contains ERBB-1 (EGFR), ERBB-2, ERBB-3 and ERBB-4 enzymes and their homo- and etero-dimers; unfortunately the oligonucleotide array does not contain spots for ERBB-2. Thus, we decide to analyze only a part of the pathway containing 29 gene products. In case of an enzyme containing multiple genes, we follow the strategy described in the previous paragraph.

### Pathways conversion

**Figure 3:**
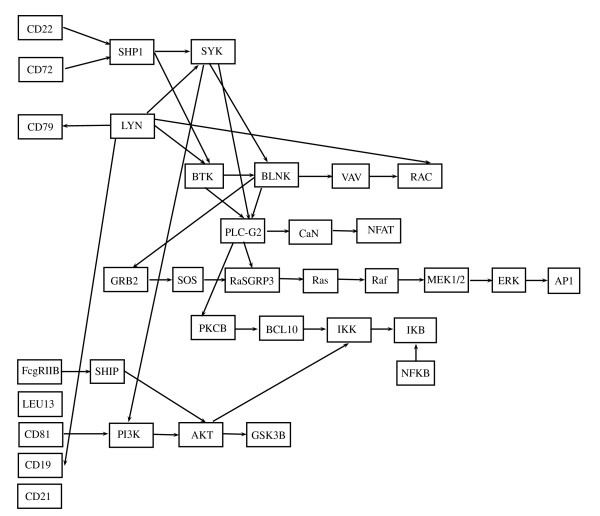
**DAG *D *obtained from BCR pathway**. DAG *D *corresponding to the BCR signaling pathway.

**Figure 4:**
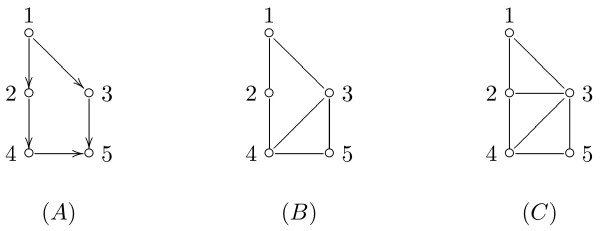
**DAG, moral graph, triangulated graph**. Example of a DAG (A), the corresponding moral graph (B), and one possible triangulated graph (C).

**Figure 5:**
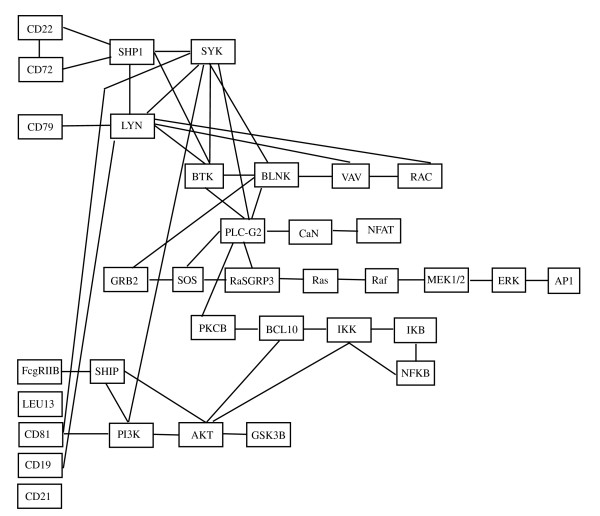
**Moral graph *D^m ^*obtained from BCR pathway**. Moral graph *D^m ^*corresponding to the BCR signaling pathway.

The pathway is converted into a graphical model following the steps described in the Methods section. Firstly, it is converted into a directed acyclic graph (DAG) *D*. For the BCR signaling pathway, for example, the DAG is shown in Figure 3. Starting from *D*, we derive its moral graph *D^m^*. An example of a moralization of a DAG is given in Figure 4. The moral graph corresponding to the DAG in Figure 3 is shown in Figure 5. In the following, we denote the undirected graph *D^m ^*with *G*. We assume to model the data in the two experimental conditions with two graphical Gaussian models [15] with the same undirected graph *G*,

$$\begin{array}{l} {ℳ}_{1}(G)\;=\;\{Y\sim N_{p}(\mu_{1},\;\Sigma_{1}),\;\Sigma_{1}^{-1}\in S^{+}(G)\},\\ {ℳ}_{2}(G)\;=\;\{Y\sim N_{p}(\mu_{2},\;\Sigma_{2}),\;\Sigma_{2}^{-1}\in S^{+}(G)\}, \end{array}$$

respectively, with *p *equal to the number of genes (vertices of the graph), see Methods for more details. All the analyses have been performed using R statistical software, with packages 'gRbase' for manipulation of graphs, 'qpgraph' for the IPS algorithm, 'samr' for the SAM test. All the R scripts are available upon request.

## Results

We are interested in (i) comparing the strength of the links between genes in two experimental conditions; (ii) testing the differential expression of the pathway. In a graphical Gaussian models context, (i) is simply achieved by comparing the two concentration matrices (inverse of the covariance matrices), because they contain all the information about the underlying structure of conditional independences among variables. Therefore, the null hypothesis to be tested is $H_{0}:\Sigma_{1}^{-1}=\Sigma_{2}^{-1}$, which, of course, is equivalent to the hypothesis Σ_1 _= Σ_2_. Testing the differential expression of the pathway is achieved by checking equality of means, i.e, by testing *H*_0 _: *μ*_1 _= *μ*_2_. Such test has a different structure according to whether the two graphical Gaussian models ℳ_1_(*G*) and ℳ_2_(*G*) are homoschedastic, i.e. they have the same covariance matrix, or not. Therefore, testing the differential expression of the pathway requires to preliminarily take a decision on equality of the two concentration matrices. All technical details are derived in Methods.

### BCR signaling pathway

The test in (1) rejects the null hypothesis (*p-value *= 0.003) for the graph shown in Figure 5; this means that the strength of the connections among genes in this pathway is significantly different in the BCR/ABL positive and BCR/ABL negative samples. This result seems to support our conjecture that in different experimental conditions the degree of connection among pathway nodes can change. Taking into account the non-homogeneity of the covariances in the two experimental conditions, we can now properly perform the test on the means reported in (2), rejecting the null hypothesis (*p-value *= 0.0001). Even if not directly comparable, this last result is in agreement with the results derived from application of the multivariate approaches of [2] (*p-value *= 0.0008) and of [8] (*p-value *< 0.0001).

Thus, patients with BCR rearrangement show a significant deregulation of the entire pathway centered on the BCR gene products with respect to those without rearrangement. BCR pathway is composed by 35 enzymes and is characterized by three ways in and four ways out. As shown before, a classical multivariate approach permits to identify the whole pathway as significantly involved in the pathology. Nevertheless, it is not able to discover the presence of possible preferential signals within the pathway. The use of graphical models in the context of gene sets analysis allows the decomposition of the moral (or, if necessary, triangulated) graph into cliques (see Methods). When tested separately, such decomposition could highlight those signals that, within the pathway, are mostly involved in its deregulation.

**Figure 6:**
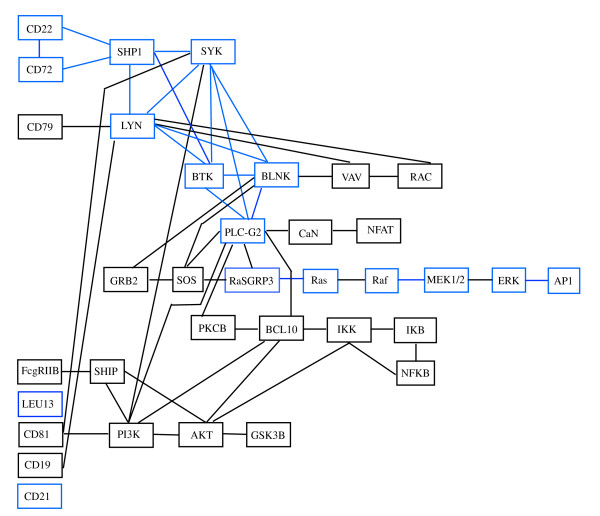
**Triangulated graph *D^t ^*obtained from BCR pathway**. Triangulated graph *D^t ^*corresponding to the BCR signaling pathway.

Since the graph *D^m ^*is not decomposable, we consider a possible triangulated graph. An example of triangulation of a graph is shown in Figure 4. The graph *D^t ^*corresponding to our pathway is shown in Figure 6, and it has five added edges and three more cliques in comparison with *D^m^*. In particular, the graph *D^t ^*has 30 cliques, whose composition is reported in Table 1. In the same table, the *p-values *of the test in (1) performed on each clique are shown in the first column. Among all the cliques, nine cliques were found with different covariance matrices in the two experimental conditions (*p-values*≤ 0.1); the cliques are displayed in blue in Figure 6. The test for differential expression repeated on each clique gives 13 cliques as differentially expressed (*p-values*≤ 0.1). The *p-values *are reported in the second column of Table 1. Note that exact tests can be performed on cliques with homogeneous covariances: the exact *p-values *are highlighted in italics.

**Table 1:** Cliques of *D*^*t *^with corresponding p-values for BCR pathway

	***H***_**0 **_: **Σ**_**1 **_**= Σ**_**2**_	***H***_**0 **_**: *μ***_**1 **_**= *μ***_**2**_
***Clique***	***p-value***	***p-value***

(PLCG2, SYK, BTK, BLNK)	0.056	0.122
(PLCG2, SYK, PI3K)	0.164	*0.075*
(PLCG2, CaN)	0.988	*0.023*
(PLCG2, SOS, BLNK)	0.570	*0.008*
(PLCG2, SOS, RaSGRP3)	0.715	*0.013*
(PLCG2, PKCB, BCL10)	0.942	*0.002*
(PLCG2, BCL10, PI3K)	0.796	*0.059*
(CD72, CD22, SHP1)	0.026	0.000
(LYN, SYK, BTK, BLNK)	0.008	0.549
(LYN, SYK, BTK, SHP1)	0.000	0.475
(LYN, CD79)	0.832	*0.780*
(LYN, CD19)	0.989	*0.358*
(LYN, VAV, BLNK)	0.920	*0.696*
(LYN, VAV, RAC)	0.336	*0.141*
(NFAT, CaN)	0.529	*0.002*
(GRB2, BLNK, SOS)	0.821	*0.201*
(Ras, RaSGRP3)	0.044	0.121
(Ras, Raf)	0.243	*0.182*
(Raf, MEK1/2)	0.101	0.825
(MEK1/2, ERK)	0.252	*0.593*
(ERK, AP1)	0.028	0.026
(IKK, BCL10, AKT)	0.430	*0.524*
(IKK, IKB, NFKB)	0.547	*0.002*
(AKT, PI3K, BCL10)	0.476	*0.142*
(AKT, PI3K, SHIP)	0.558	*0.215*
(AKT, GSK3B)	0.631	*0.017*
(CD81, SYK, PI3K)	0.395	*0.193*
(SHIP, FcgRIIB)	0.980	*0.443*
LEU13	0.103	0.003
CD21	0.008	0.008

Cliques of the triangulated graph *D^t ^*with corresponding *p-values *for the BCR pathway. The first column refers to the test (1), the second one to the test (2). The *p-values *in italics in the second column are exact, i.e., are computed on the exact null distribution of the test statistics.

Interestingly, we found that only few cliques are characterized by significant *p-values *in both tests, some show only significant test (1) and some others significant test (2). In particular, cliques resulted to be significant by means of test (1) identify a clear path starting from CD22 and CD72 and ending at AP1 (also known as JUN, jun oncogene), going through RasGRP3, Ras, Raf, MEK1/2 and ERK enzymes (both involved in the MAPK signaling pathway, see Figure 1). The starting and the ending point of this path is composed by cliques with both tests significant. This path is in agreement with the experimental findings about BCR/ABL fusion gene consequences. In particular, expression of BCR/ABL leads to activation of Ras. The events subsequent to Ras activation suggest an involvement of MAPK and JNK pathways leading to an activation of JUN [16,17].

On the other hand, cliques resulted to be significant by means of test (2) seem to be more sparse along the pathway. They identify the way in (CD72, CD22, SHP1) and the three ways out, (NFAT, CaN), (ERK, AP1) and (IKK, IKB, NFKB) of the pathway. These results are concordant with recent findings that show the involvement of NFAT and NFKB signaling in leukemia development. Deregulation of calcineurin/NFAT signaling and/or abnormal expression of its components, have recently been reported in solid tumors of epithelial origin, lymphoma and lymphoid leukemia. In mouse models of human T-ALL/lymphoma the persistent activation of calcineurin/NFAT signaling is shown to be pro-oncogenic, [18]. Moreover, NFKB is a transcription factor that regulates genes involved in immune and inflammatory responses, cell proliferation, and cell differentiation and has recently been shown to be antiapoptotic. BCR/ABL signaling leads, through Ras, to an increase in NFKB-dependent gene expression [19]. All these signal cascades lead to cell proliferation, a biological process that is known to be highly involved in tumor development and progression.

We remark that, in testing the hypotheses on the cliques, there is the possibility that some cliques will be declared statistically different at some significance level, even if the corresponding null hypotheses are true (type I errors). Given the significance level for the tests, the proportion of type I errors in the 30 test is difficult to estimate, because the test statistics used to perform the single tests are not independent, being functions of overlapping sets of variables. With reference to the BCR pathway, we studied this question via a simulation study. For 1000 runs, we generated samples for the two conditions (BCR-presence of rearrangement, NEG-absence of rearrangement) from the same graphical model ℳ(*G*) = {*Y *~ *N*_35_(*μ*, Σ), Σ^-1 ^∈ *S*^+^(*G*)}, having the undirected graph *G *associated to the BCR pathway. Decomposition of such model clearly leads to the same cliques previously studied; obviously, all the 30 hypotheses relative to equality of the covariances (means) on the cliques are, in this setting, true by construction. At each run, we counted the number *m*, 0 ≤ *m *≤ 30, of rejections (type I errors) observed in the 30 tests. The distribution of *m *gives an indication on the proportion of type I errors on the 30 cliques that one should expect.

Different initial configurations, in terms of values of the mean *µ*, of the covariance matrix Σ, and of sample sizes, were tried. Here, we discuss the results for a setting reproducing our experimental conditions. We set *n*_1 _= 37 (BCR) and *n*_2 _= 41 (NEG), with an initial covariance matrix Σ equal to the sample estimate of the matrix in the NEG condition. Without loss of generality, we fixed *μ *= 0. The mean number of cliques erroneously rejected was 1.5, and the proportion of cases with more than nine rejections is 0.001, suggesting that it is extremely rare observing a number of rejections greater than the number that we have observed on our experimental data when all the null hypotheses are true.

### ERBB signaling pathway

**Figure 7:**
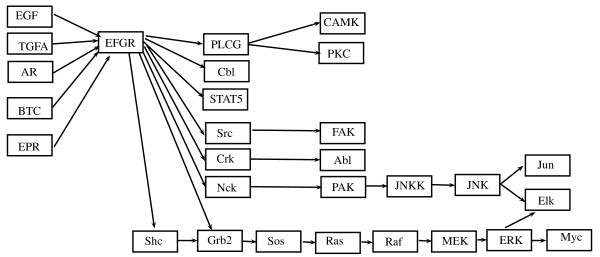
**DAG *D *obtained from ERBB pathway**. DAG *D *corresponding to the chosen portion of ERBB signaling pathway.

**Figure 8:**
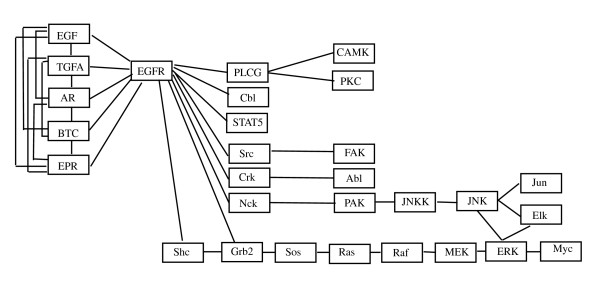
**Moral graph *D^m ^*obtained from ERBB pathway**. Moral graph *D^m ^*corresponding to the chosen portion of ERBB signaling pathway.

The same analyses have been performed on a part of the ERBB signaling pathway (see the corresponding DAG in Figure 7). Firstly, we found that the test in (1) rejects the null hypothesis (*p-value *= 2.3 × 10^-8 ^) for the moral graph shown in Figure 8; thus the strength of the connections among genes in this pathway is significantly different between EGFR positive and negative mutated samples. The test on the means reported in (2) rejects the null hypothesis (*p-value *= 0.0045). This last result is in agreement with the results derived from application of the multivariate approaches of [2] (*p-value *= 0.0659) and of [8] (*p-value *= 0.0037).

**Figure 9:**
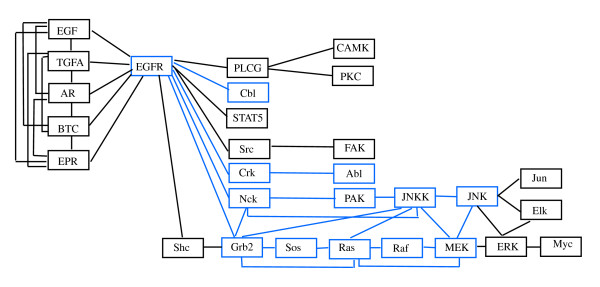
**Triangulated graph *D^t ^*obtained from ERBB pathway**. Triangulated graph *D^t ^*corresponding to the chosen portion of ERBB signaling pathway.

After triangulation of the moral graph (see Figure 9), the part of the EGFR pathway we considered is composed by 23 cliques (as reported in Table 2), five cliques as input, all coupling with EGFR gene, and three cliques as output (JNK-Jun, JNK-Elk-ERK and ERK-Myc). The test in (1) performed on each single clique identifies 11 cliques with significantly different covariance matrices (*p-value *≤ 0.05) in the two groups of patients. The cliques are highlighted in Figure 9. The test for differential expression gives only six cliques as differentially expressed (*p-value *≤ 0.05, see Table 2). As in the previous case, only a small number of cliques show significant *p-value*s in both tests (1) and (2). According to results of test (1) there are two clear signal paths in the graph starting from EGFR gene following MAPK pathway ending respectively to ERK and JNK. Both paths lead to cell proliferation and differentiation, two key biological processes in cancer development. These results are in agreement with experimental findings that show how EGFR mutations result in activation of the antiapoptotic pathways (PI3K/AKT and JAK-STAT), and in cellular proliferation through ERK/MAPK signaling [20]. A simulation study similar to the one performed for the BCR pathway has been conducted, showing that proportion of cases in which more than 11 null hypotheses are rejected is 0.001.

**Table 2:** Cliques of *D*^*t *^with corresponding p-values for the ERBB pathway

	***H***_**0 **_**: Σ**_**1 **_**= Σ**_**2**_	***H***_**0 **_**: *μ***_**1 **_**= *μ***_**2**_
***Clique***	***p-value***	***p-value***

(EGFR, EGF, TGFA, AR, BTC, EPR)	0.245	*0.147*
(EGFR, PLCG)	0.233	*0.006*
(EGFR, Cbl)	0.004	0.007
(EGFR, STAT5)	0.141	*0.318*
(EGFR, Src)	0.224	*0.176*
(EGFR, Crk)	0.047	0.196
(EGFR, Nck, Grb2)	0.018	0.104
(EGFR, Shc, Grb2)	0.390	*0.159*
(Sos, Grb2, Ras)	0.031	0.145
(Ras, MEK, Raf)	0.022	0.030
(Ras, MEK, JNKK)	0.004	0.694
(Ras, Grb2, JNKK)	0.048	0.757
(MEK, JNK, JNKK)	0.009	0.186
(MEK, JNK, ERK)	0.678	*0.075*
(ERK, JNK, Elk)	0.125	*0.242*
(ERK, Myc)	0.515	*0.101*
(Src, FAK)	0.421	*0.524*
(Crk, Abl)	0.000	0.135
(PAK, Nck, JNKK)	0.004	0.099
(JNKK, Nck, Grb2)	0.011	0.190
(JNK, Jun)	0.559	*0.033*
(PLCG, CAMK)	0.930	*0.012*
(PLCG, PKC)	0.127	*0.048*

Cliques of the triangulated graph *D^t ^*with corresponding *p-values *for the ERBB pathway. The first column *p-values *refers to the test (1), the second one to the test (2). The *p-values *in italics in the second column are exact, i.e., are computed on the exact null distribution of the test statistics.

### Testing single vertices

It is worth studying also the significance of a clique in relation to its individual members. It is not necessarily true that all members of a significant clique are, individually, statistically significant. Table 3 reports the *q-values *[21] from the SAM test [13] performed on each gene of the BCR signaling pathway. For example, the clique (LYN, SYK, BTK, BLNK) has a significant *p-value *but neither of the single genes has a significant *q-value*. Table 4 reports the same quantities for each gene of the ERBB signaling pathway. In this case, *q-values *associated to ERBB pathway nodes show a moderate significance (the lowest *q-value *is 0.09); this demonstrates how a gene set approach using signaling pathways, and in particular, our graphical approach is able to improve microarray results interpretation.

**Table 3:** q-values from SAM analysis for each gene of the BCR pathway

*Gene*	*q-value*
CD22	0.00
CD72	0.00
SHP1	0.17
SYK	0.41
CD79	0.55
LYN	0.41
BTK	0.17
BLNK	0.50
VAV	0.50
RAC	0.13
PLCG2	0.09
CaN	0.00
NFAT	0.00
GRB2	0.41
SOS	0.00
RaSGRP3	0.44
Ras	0.17
Raf	0.50
MEK1/2	0.41
ERK	0.50
AP1	0.00
PKCB	0.05
BCL10	0.55
IKK	0.55
IKB	0.00
NFKB	0.00
FcgRIIB	0.41
SHIP	0.29
PI3K	0.00
AKT	0.17
GSK3B	0.00
CD19	0.09
CD21	0.00
CD81	0.41
LEU13	0.00

Genes of the BCR signaling pathway with corresponding *q-values *[21] from SAM analysis [13].

**Table 4:** q-values from SAM analysis for each gene of the ERBB pathway

*Gene*	*q-value*
EGF	0.29
TGFA	0.09
AR	0.09
BTC	0.09
EPR	0.09
EGFR	0.22
PLCG	0.09
Cbl	0.09
STAT5	0.20
Src	0.20
Crk	0.24
Nck	0.09
CAMK	0.24
PKC	0.29
FAK	0.23
Abl	0.09
PAK	0.09
JNKK	0.20
JNK	0.09
Jun	0.09
Elk	0.16
Shc	0.09
Grb2	0.20
Sos	0.09
Ras	0.13
Raf	0.11
MEK	0.16
ERK	0.22
Myc	0.09

Genes of the ERBB signaling pathway with corresponding *q-values *[21] from SAM analysis [13].

As a general view, it seems that pathological conditions are able to alter not only gene expression but also the strength of the connection among genes. These results partially change the idea that pathway edges are static entities, moving towards the concept that links among gene products modify their strength according to the molecular modifications. Thus, in order to have an overall idea of the pathway deregulation resulting from the pathology investigated, we suggest that the complete information derived from tests (1) and (2) should be used, whenever gene sets analysis wants to be performed.

## Conclusions

Multivariate approaches proposed so far for gene sets analysis test, through gene expression values, pathways mean differences between groups. However, mean differences are related to correlation differences among genes within a gene set. Here, we propose two tests that exploit the graphical evidence of a pathway, and allow the decomposition of the pathway into components that can be marginally compared. The first one is focussed on the strength of the links among genes of a pathway between two groups. The second test is the usual test for differential expression, but in addition it contains the information coming from the topology of the pathway. This permits to evaluate in detail which components of the pathway contribute at most in its deregulation. It is worth noting that the components of a biological pathway are not always regulated at the level of expression, but at the level of proteins. Therefore other types of data may be useful to detect changes between proteins in a pathway.

Our approach is tested on gene expression data on i) acute lymphocytic leukemia (ALL) with and without BCR/ABL gene rearrangement and ii) lung adenocarcinoma with and without EGFR mutation. In the first case our approach identifies, as expected, the B cell receptor pathway significantly involved in groups difference and shows that only a part of the entire pathway seems to be responsible of the different prognostic behaviour of BCR/ABL positive and negative patients. In particular, we find, in agreement with published experimental evidences, that JUN oncogene with RAS/MAPK/JNK followed by NFAT and NFKB seem to be the key regulatory elements in the comparison of BCR/ABL positive and negative patients.

In the second case, the EGFR pathway is found significantly involved in the difference between lung cancer patients with and without EGFR mutation. However, within EGFR pathway we found significant only two ways through the MAPK pathway leading to proliferation and differentiation processes.

Pathway sizes are highly heterogeneous, there are pathways characterized by complex structures, and, then, composed by more than fifty gene products, and other composed by less than ten gene products. From a statistical point of view, inference results could be affected by the size of the pathway. However, the idea of focussing on components of a pathway may be particularly useful also for the inference aspects of the *n *<*p *cases. When the sample size is smaller than the number of genes, i.e. *n *<*p*, and, in addition, *n *is smaller than the dimension of the larger clique of the graph, a shrinkage covariance matrix with null elements corresponding to the missing edges of the graph *G *may be employed. This is a modification of the approach proposed by [8], where a shrinkage covariance matrix [22] is used as estimate of the covariance matrix employed by the test. In our context, the same shrinkage covariance matrix must be used as input of the IPS algorithm. In this way, the estimated covariance matrix is a covariance matrix of a Gaussian graphical model with graph *G*. If a shrinkage estimator for the covariance matrices is employed, permutation methods have to be applied to derive an approximation of the null distribution of the test statistic -2 log Λ. Most of the time, researchers do not know which pathway to investigate in the first place. Or scientists have an interest in many pathways, because most conditions are the outcome of effects in more than one pathway and some pathways may get affected more than others. Just because a pathway changes moderately, it would not necessarily mean that the condition is an outcome of that. Which pathway(s) to focus upon is a very critical decision that should be taken by experts on the basis of the research questions. We remark that our technique allows to check for differences on each of such pathways, so that a global picture could be composed by composition of the results on the individual pathways.

## Methods

### Converting a pathway into a graphical model

Even if there is not a precise definition, a biological pathway can be described as a set of linked biological components interacting with each other over time to generate a single biological effect. They comprise a myriad of interactions, reactions, and regulations, which are often identified piecemeal over extended periods and by a variety of researchers. Moreover, participants in one pathway can be involved also in others, leading to dependent pathways. As a result, the pathway's topology has to be considered as a dynamic entity whose information is particularly challenging to compile and organize. There are several valid and exhaustive pathway repositories reporting pathway topologies as networks of functional interactions such as KEGG [9], Biocarta, Reactome [23] and WikiPathways [24].

In this study, we use KEGG maps, as they represent a good compromise between map accurateness and simplicity. Nevertheless, it is important to note that our approach can be used whichever is the origin of the map. KEGG [9] is one of the mostly used pathways databases, with more than a hundred of pathways and more than fifty available signaling pathways. Figure 1 represents an example of such pathways, the B cell receptor signaling pathway in human. It is composed by edges and nodes, which have the following meanings. Rectangles represent gene products, mostly proteins, but also RNA and complexes. The edges between rectangles represent functional interactions and they can be undirected, directed, directed with a +p, directed with a -p, dashed and directed, directed with a bar at the end. Circles are other types of molecules, mostly chemical compounds, while the large white rectangles are the links to other pathways. See the KEGG website for a more detailed description and meaning of the different types of edges and circles.

We refer the reader to the Appendix for an essential lexicon on graphical models. To keep track of the initial structure of the genes, we convert the structure of a pathway into a directed acyclic graph (DAG) *D*, by following these simple steps: i) inhibition, phosphorylation (+p) and dephosphorylation (-p) are considered as simple directed edges; ii) undirected edges are directionated using additional information derived by Biocarta pathway and iv) in case of complexes (nodes composed by multiple gene products) we consider as expression of the complex the first principal component. This is a linear combination of gene expression values of all gene products in the same complex which retains most of the variation in the dataset.

Then, we convert *D *into a moral graph, *D^m^*, by adding edges between the parents of each vertex (if not already present), and, then, by removing the directionality of the original edges (see the Appendix). Usually, *D^m ^*has more edges than *D*, but the choice of working with a moral graph does not affect the purpose of this study. We assume to model the data of the same pathway in different experimental conditions as realizations of undirected graphical Gaussian models [15] sharing the same undirected graph *G *given by the moral graph *D^m^*. To exemplify, in the case of two conditions, we assume the Gaussian models

$$\begin{array}{l} {ℳ}_{1}(G)\;=\;\{Y\sim N_{p}(\mu_{1},\;\Sigma_{1}),\;\Sigma_{1}^{-1}\in S^{+}(G)\},\\ {ℳ}_{2}(G)\;=\;\{Y\sim N_{p}(\mu_{2},\;\Sigma_{2}),\;\Sigma_{2}^{-1}\in S^{+}(G)\}. \end{array}$$

Here, *p *is the number of genes (vertices of the graph) and *S*^+^(*G*) is the set of symmetric positive definite matrices with null elements corresponding to the missing edges of *G*. Note that the assumption of normality of the data is motivated by the well known fact that relative or absolute gene expression measurements are approximately normal on the log scale.

Of course, in real applications, the parameters of the models, i.e. the means *μ*_1 _and *μ*_2 _and the covariance matrices Σ_1 _and Σ_2 _are not known, although the position of zeros elements in the concentration matrices $\Sigma_{1}^{-1}$ and $\Sigma_{2}^{-1}$ is defined by the graph. Therefore, they need to be estimated from the data. The estimates of the covariance matrices can be obtained by running the Iterative Proportional Scaling algorithm (IPS in the following, see [15]) on the sample covariance matrices, which guarantees that the estimated matrices are positive definite and their inverse have null elements corresponding to the missing edges of the graph. The sample covariance matrices can be computed by selecting the expression levels of the genes on the pathway and by computing the sample covariances. The same matrices can be obtained from the chip covariance matrices by extracting the elements corresponding to all the pairs of genes in the pathway.

### Comparing gene sets

Adopting the graphical arguments, the assessment of whether the expression of a pathway changes in different experimental conditions fits naturally within the framework of testing hypothesis on the graphical models. The process of identifying the null hypotheses to be tested is now particularly simple.

Several potential reasons may exist inducing differences in the global expression of the pathway. A possibility is that the expression changes because the strength of the relations defining the pathway change in different conditions. As information on the relations is stored into the covariance matrices of the graphical models, such hypothesis corresponds to the hypothesis of homogeneity of the covariance matrices. Moreover, the mean expression may change, irrespective to what happens to the correlations among genes. The corresponding hypothesis is that of equality of two means. Despite the deceiving simplicity of this hypothesis, its assessment is not trivial and depends on whether the covariances of the models, generally unknown, are homogeneous or not. Therefore, an appropriate analysis of the means depends on the decision taken on the homogeneity hypothesis. In what follows, we will describe such tests, following the ideal sequence in which we think that they should be performed on real data. It is worth noting that normal distributions are fully characterized by the first two orders of moments. Therefore, tests covering the first two moments fully exploit the distributions' characteristics. We will assume to have two experimental conditions.

#### Testing equality of the strength of the relations among genes

We are interested in comparing the strength of the links between genes in two experimental conditions. In a graphical Gaussian models context this is simply achieved by comparing the two concentration matrices (inverse of the covariance matrices), because they contain all the information about the underlying structure. Therefore, the interest is in testing the hypothesis $\Sigma_{1}^{-1}=\Sigma_{2}^{-1}$.

The following methodology is the transposition of the methods for comparing covariance matrices [25] to the specific case of graphical Gaussian models.

Without loss of generality, suppose to have $y_{1}=(y_{1}^{j})$, *j *= 1, ⋯, *n*_l _observations from *N_p_*(0, Σ_1_) and $y_{2}=(y_{2}^{j})$, *j *= 1,⋯, *n*_2 _observations from *N_p_*(0, Σ_2_), with $\Sigma_{1}^{-1}=K_{1}\in S^{+}(G)$ and $\Sigma_{2}^{-1}=K_{2}\in S^{+}(G)$.

We want to test the hypothesis

(1) $$H_{0}:K_{1}=K_{2}\;\text{against}\;H_{1}:K_{1}\neq K_{2}.$$

If we set $W_{i}=\sum_{j=1}^{n_{i}}\left(y_{i}^{j}\right){\left(y_{i}^{j}\right)}^{T}$, *i *= 1, 2, the likelihood function, *L*(*K*_1_, *K*_2_) is

$$L(K_{1},\;K_{2})=\prod_{i=1}^{2}{\left(2\pi \right)}^{-\frac{n_{i}p}{2}}{\left(\det K_{i}\right)}^{\frac{n_{i}}{2}}e^{-\frac{1}{2}\text{tr}(K_{i}W_{i})},$$

and each block of it may be maximized separately [25]. In more detail, the estimates ${\hat{K}}_{1}$ and ${\hat{K}}_{2}$ are computed by direct calculation (if the graph is decomposable) or, in general, by using the IPS algorithm, see [15]. Under the null hypothesis, the algorithm computes the estimate, $\hat{\Sigma }$, of the common covariance matrix Σ starting from the pooled covariance matrix

$$S={\left(n_{1}+n_{2}-2\right)}^{-1}\cdot \{(n_{1}-1)\cdot \;S_{1}+(n_{2}-1)\cdot S_{2}\}.$$

Under the alternative hypothesis, the algorithm computes ${\hat{\Sigma }}_{1}$ and ${\hat{\Sigma }}_{2}$ using the sample covariance matrices

$$S_{1}={\left(n_{1}-1\right)}^{-1}\cdot \;W_{1}\;\text{and}\;S_{2}={\left(n_{2}-1\right)}^{-1}\cdot \;W_{2}.$$

Let ${\hat{K}}_{1}={\left({\hat{\Sigma }}_{1}\right)}^{-1}$, ${\hat{K}}_{2}={\left({\hat{\Sigma }}_{2}\right)}^{-1}$, $\hat{K}={\left(\hat{\Sigma }\right)}^{-1}$. The likelihood ratio test, Λ, is

$$\lambda =\frac{L_{H_{0}}({\hat{K}}_{1},{\hat{K}}_{2})}{L_{H_{1}}({\hat{K}}_{1},{\hat{K}}_{2})}=\frac{L_{H_{0}}(\hat{K})}{L_{H_{1}}({\hat{K}}_{1},{\hat{K}}_{2})}.$$

If we let *W *= *W*_1 _+ *W*_2_, and exploit the fact that $\text{tr}({\hat{K}}_{i}W_{i})=n_{i}\text{tr}({\hat{K}}_{i}{\hat{K}}_{i}^{-1})=n_{i}p$ and $\text{tr}(\hat{K}W)=(n_{1}+n_{2})\text{tr}(\hat{K}{\hat{K}}^{-1})=(n_{1}+n_{2})p$ (see [15]), we have

$$\lambda =\prod_{i=1}^{2}{\left(\frac{\det\hat{K}}{\det{\hat{K}}_{i}}\right)}^{\frac{n_{i}}{2}},$$

and

$$-2\log\lambda =\sum_{i=1}^{2}n_{i}\log\left(\frac{\det{\hat{K}}_{i}}{\det\hat{K}}\right).$$

The asymptotic null distribution of -2 log Λ is $\chi_{r+p}^{2}$, where *r *is the number of edges of *G*. The above given test might suffer of a weakness. It is well known from the literature that the likelihood ratio test for the equality of two covariance matrices is not particularly robust to violations of the normality assumption. If this is the case, several robust variants of the above given test could be defined following the developments already available in the literature. For example, one could follow [26], who proposes several Wald tests for elliptical and non-normal distributions.

If the test rejects the null hypothesis, the graphical approach allows us to look for the sources of differences of the two concentration matrices. In fact, if the graph is decomposable, it is possible to decompose it into its maximal complete subgraphs (cliques) and repeat the previous test for each clique. In this way, the maximum likelihood estimate of the sub-covariance matrix for the variables belonging to the same clique is directly performed with the available data on the cliques and there is no need of any marginalization. The test of equality of the covariance matrices on the cliques can be tested by following the standard methods (see [25]) without need of using the results described at the beginning of this section. Note that, in this way, the cliques are compared only marginally. If the graph is not decomposable, it is always possible to add extra edges in order to obtain a new graph that is triangulated and therefore decomposable (see the Appendix for more details). The test can then be performed on the cliques of this graph.

#### Testing for differential expression

If the null hypothesis in (1) is not rejected, the differential expression of the pathway is tested by the hypothesis

$$H_{0}:\mu_{1}=\mu_{2}\;\text{subject}\;\text{to}\;\Sigma_{1}=\Sigma_{2}.$$

This test can be performed by using exact procedures such as a multivariate analysis of variance (MANOVA), as, for example, in [8]. Otherwise, if we reject the null hypothesis of homogeneity in (1), the hypothesis to be tested is

(2) $$H_{0}:\mu_{1}=\mu_{2}\;\text{subject}\;\text{to}\;\Sigma_{1}\neq \Sigma_{2}.$$

This is the usual test for equality of means in a two sample problem with unequal covariance matrices, also called Behrens-Fisher problem (see [25]).

In both cases, the standard tests are to be opportunely adjusted in order to consider that the data come from Gaussian graphical models, and, therefore, have a structured covariance matrix. This, clearly, involves a constrained estimation of the covariance matrices, carried out, for example, with the IPS algorithm.

## Appendix

In this section, we concisely collect some definitions about graphs and their properties that are useful for reading the paper. For more details, see [15].

A graph *G *is a pair *G *= (*V*, *E*), where *V *is a finite set of vertices and the set of edges *E *⊆ *V*×*V *is the set of ordered pairs of distinct vertices. If both (*u*, *v*) ∈ *E *and (*u*, *v*) ∈ *E*, the edge (*u*, *v*) is said to be undirected. If (*u*, *v*) ∈ *E *but (*u*, *v*) ∉ *E*, the edge (*v*, *u*) is said to be directed.

If the graph has only undirected edges, it is called undirected, whereas if all the edges are directed, it is called directed. In an undirected graph, if there is an edge between *u *and *v*, *u *and *v *are said to be adjacent. In a directed graph, if *u *→ *v, u *is said to be a parent of *v *and *v *a child of *u*.

A path is a sequence of vertices such that every vertex has an edge to the next vertex in the sequence. A cycle is a path such that the first vertex of the path corresponds to the last one.

A directed acyclic graph (DAG) is a directed graph without cycles. Given a DAG *D*, a moral graph *D^m ^*is the undirected graph obtained from *D *by adding undirected edges between all pairs of vertices that have a child in common (if they are not already present) and then by rendering all edges undirected.

A graph is complete if *E *contains all pairs of distinct elements of *V*. *G_A _*= (*A*, *E_A_*) is a subgraph of *G *= (*V*, *E*) if *A *⊆ *V *and *E_A _*= *E *⋂ (*A*×*A*). A complete subgraph that is not contained within another complete subgraph is a clique.

A triple (*A*, *B*, *C*) of disjoint subsets of *V *of an undirected graph *G *is a decomposition of *G *if *V *= *A *⋃ *B *⋃ *C*, *C *is a complete subset of *V *and *C *separates *A *and *B*. An undirected graph is decomposable if either it is complete or it possesses a proper decomposition (*A*, *B*, *C*) such that both subgraphs *G*_*A*⋃*B *_and *G*_*B*⋃*C *_are decomposable.

A triangulated graph (also called chordal graph) is an undirected graph with the property that every cycle of length *n *≥ 4 has two non-consecutive vertices that are adjacent.

An important result is that an undirected graph is decomposable if and only if it is triangulated, see [15].

## Authors' contributions

All Authors jointly developed the methods and wrote the paper. All Authors read and approved the final manuscript.
